# Between normative universality and sharing embodied knowledge–exploring the (re-) definition of legitimate knowledge and knowers using the example of the German public media debate about racism

**DOI:** 10.3389/fsoc.2025.1536195

**Published:** 2025-04-23

**Authors:** Ana-Nzinga Weiß

**Affiliations:** Institut für Medienforschung, Philosophische Fakultät, Universität Rostock, Rostock, Germany

**Keywords:** racism, media representation, social inequality, hybrid media system, social media, social epistemology, knowledge, critical discourse analysis

## Abstract

This article seeks to present a new approach to studying the dynamics of constructing legitimate knowledge and speaker positions in public media discourse that is characterized by a hybrid media system. The basic framework of this approach is built on the paradigm of social epistemology and the presumption that although knowledge can be shared, the conditions of sharing are subject to social power structures. By conceptualizing the media as part of social epistemological processes, I offer a conceptual innovation that allows for a nuanced and critical analysis of how inequalities can be (re)produced in media representation. Within the context of the German public debate about racism since the Black Lives Matter protests in the summer of 2020, I analyzed talk shows on the topic of racism that aired on German public television, YouTube, and Instagram by means of a Critical Discourse Analysis as a means of illustrating analysis within this framework. My analysis revealed three patterns through which it was possible to construct legitimate knowledge and speaker positions about racism: (1) performances of a rational and equitable exchange of opposing epistemic positions, (2) performances of counter-hegemonic positionality in communal exchange, and (3) performances of a rational exchange of embodied knowledge. The results illustrate the delicate interplay of different power structures within the construction of knowledge regarding racism. I conclude with an emphasis on the need for a parrhesian praxis in social analysis in service of being constantly self-critical and, at the same time, critical of power.

## Introduction

“[…] I do not want to explain whether racism really exists – bullshit! […] I do not need some TV channel to give me a stage. I already have my stage, you know. All you have to do is lift the curtain, I’m already here […]” (Tesfu, 2020 [W], at 31:22–32:59, author’s translation).

In this statement, Black German content creator Tarik Tesfu expresses his dissatisfaction with the German public-service broadcasting system’s approach to discussing racism in the summer of 2020. The summer of 2020 marked a pivotal moment in Germany’s public engagement with racism. Germany has long been hesitant to openly discuss racism, and, compared to other nations, it has conducted relatively little research on this societal phenomenon (Çaglar and Sridharan, 2021, pp. 61–62; see also Salem and Thompson, 2016). This public disengagement was interrupted in the summer of 2020 when traditional mass media responded to the global Black Lives Matter (BLM) protests following the killing of George Floyd (Haruna-Oelker, 2020).

However, soon discussions arose in response to German traditional media, especially on social media platforms like Twitter (now X), concerning the alleged exclusion from traditional media of people experiencing racism. Further, traditional media were accused of an ignorant attitude towards racism as the quote above illustrates. Consequently, content creators began to publish in various formats on social media platforms complementing, critiquing, or countering traditional media’s engagement. As a result, a public media debate over who can claim legitimate knowledge on the topic of racism emerged across German traditional and social media.

Spivak (2006) has theorized public struggles over legitimate speaker positions. With the question “Can the subaltern speak?” (Spivak, 2006, p. 32), she points to an epistemic hierarchy that violently excludes the knowledge of those persons who are “without lines of social mobility” (Spivak, 2006, p. 28). In social collectives characterized by unequal power dynamics, this is importantly tied to questions of representation: “Who can represent whom, when and how? To what extent is representation a violent practice? And how far can political practice function without representation?” (Castro Varela et al., 2018, p. 270).

As representers and (re-)producers of social discourses (Klaus and Kirchhoff, 2016, p. 529), the media are influential in constructing a society’s hegemonic knowledge pool (e.g., Hall, 2012, pp. 102–103). Through their politics of representation, they thus (re-)produce epistemic hierarchies. Critical scholars in media and communication, informed by feminist, critical race, or postcolonial theories, for example, have investigated media representation in relation to social structures of dominance (e.g., Said, 1979; Hall, 1997). Others have focused on epistemology in media and communication research (e.g., Ekström and Westlund, 2019; Godler et al., 2020). These approaches usually focus on researching journalism (e.g., Ekström and Westlund, 2019; Godler et al., 2020) and the journalistic ideal of objectivity (e.g., Durham, 1998; Muñoz-Torres, 2012).

The media are not only characterized by their entanglement with different societal discourses. The rise of the Internet (specifically digital networks provided by the Web 2.0) and its establishment in everyday media use has further complicated traditional distinctions made regarding media production, distribution, and use. Importantly, production is thereby not only organized within hierarchical institutions, but rather it is open to participation based on communal collaboration and without standardized quality control (Bruns, 2014, p. 3). This fluidity in roles has enabled “new formations of societal speaker positions” (Lünenborg, 2016, p. 331) that are relevant to public discourse. The resulting range of accessible information and perspectives on socially relevant topics further challenges media formats that have traditionally relied on their unique gatekeeping role. Public media communication is thus characterized by hybridity shaped by multiple actors, media formats, and logics, as well as by their corresponding discursive power relations (Chadwick, 2013).

In this article, I expand on previous research on epistemology and societal power structures in the media by focusing on public knowledge production within the context of a hybrid media system, meaning that classical journalistic media and participatory platform media coexist in a reflexive relationship. I illustrate my approach with results from a critical discourse analysis (CDA) of German public talk shows aired between 2020 and 2021 on the topic of racism. Against this backdrop, the article’s guiding research question is formulated as follows: **“**How is knowledge constructed in political talk about racism in German talk shows?”

In the following, I first introduce the paradigm of social epistemology and concepts related to the entanglement of knowledge and power, which form the theoretical foundation of this article. Following this, I conceptualize the media as part of a social epistemological process. The subsequent section provides an overview of public engagement with racism in Germany, both before and after the killing of George Floyd and the subsequent global BLM protests. Under *Materials and Methods*, I establish Critical Discourse Analysis as the method of investigation and introduce the sample used to examine the article’s research question. Thereafter, the study’s results are presented and discussed. The article concludes with a summary of its main contributions.

## Knowledge and power

“We think not only as individuals and as human beings, but also as social beings, products of particular social environments that affect as well as constrain the way we cognitively interact with the world.” (Zerubavel, 1999, p. 6).

With this statement Zerubavel (1999) describes knowledge and knowing as embedded in social interactions and social contexts. While cognitive science exploring how people think, make sense of the world, and acquire knowledge and truth has long regarded cognition as an individual process, it is, in fact, influenced by the social matrices in which people find themselves (Zerubavel, 1999, pp. 5–6). The conditions under which knowledge is produced are examined by the philosophical paradigm of epistemology. In other words, epistemology is concerned with the “study of knowledge and truth,” or as Gunzenhauser and Gerstl-Pepin (2006) specify, it “is a theory of what gets to count as knowledge” (p. 332).

It is important here to realize that human beings are not limited to their own experience in making sense of the world. By communicating with each other, knowledge is transferred and shared in different social contexts (Zerubavel, 1999, pp. 7–8). Social epistemology examines the acquisition of beliefs based on the testimony of others, the epistemic composition of knowledge-producing and distributing institutions, and the implications that social dimensions of knowledge have for our understanding of rationality, justified beliefs, and knowledge (Godler et al., 2020, p. 216).

In a social epistemology approach, knowledge in society is indivisibly entangled with power. Fricker (2017) has introduced the concept of *epistemic injustice* to describe a form of discrimination that occurs when a person is degraded or disadvantaged in their status as an epistemic subject. Epistemic injustice has become a significant concept in the study of knowledge and power (Fricker, 2017, p. 53). In addition to Fricker, a number of scholars from various backgrounds, including feminist, critical race, and decolonial philosophy, have examined how power operates within different instances of people trying to contribute their knowledge, interpretations, beliefs, and opinions to the socially shared pool of ideas (Pohlhaus, 2017, p. 13).^1^ Dotson (2012), for instance, refers to *epistemic oppression* in describing exclusions of knowers from a “given epistemic community” that inhibit them in their use of “persuasively shared epistemic resources,” (p. 24) as well as in revising those resources. The excluded subject can thus neither use nor influence a given knowledge pool. Coining the term *epistemic violence*, Spivak (1988) emphasizes the role of Western intellectual production within contemporary power relations. This concept serves to investigate the “complex silencing of marginalized groups through appropriation and homogenization” (Tuana, 2017, p. 128).

Almeida (2015) argues that even today “Eurocentrism, hegemony and colonialism (re)produce ‘legitimate’ knowledge and knowers in the Western world” (p. 79). She furthers her claim, stating that Western mainstream academia “reinscribes colonial and racial thinking by strategically reducing vast theoretical contributions of racialized or Indigenous scholars to experimental insights or ‘stories’” (Almeida, 2015, p. 79). Corresponding values and interests that underlie supposedly neutral methods in epistemology, such as Eurocentrism, androcentrism, and heteronormativity, continue to exclude people who deviate from these standards. Their knowledge is thus hidden by dominant values and interests (Tuana, 2017, pp. 125–128). Alcoff (2017) describes this exclusionary approach to knowledge as an *epistemology of ignorance.* It is important here to realize that there is nothing wrong with a Eurocentric or androcentric perspective, per se. Rather what is to be criticized and challenged is the assumption of its universality, the exclusion and delegitimation of other “modes of thinking,” which mark them as biased or untrue while making use of unequal distributions of power (Ladson-Billings, 2003, pp. 6–7), combined with the failure to realize that dominant ways of thinking are socially and politically situated as well (Harding, 2004, p. 39).

## Changing the subject of knowledge in unequal epistemic structures

Different approaches have been used to redefine the subject of knowledge in contrast to the notion of a disembodied, universal, rational individual. Theoretically, they all work to illuminate the ways in which repressive practices can produce or increase epistemic inequalities, marginalization, and exclusions. Their methodological goal is to reveal interests and values on which the supposedly neutral methods in epistemology and science are based. This also poses questions about who is regarded as a knowing subject and how particular qualities and group situations influence this status (Tuana, 2017, pp. 125–128; see also Hill Collins, 2000, p. 252). Feminist standpoint theory, for instance, focuses on knowledge that is hidden by dominant values and interests, arguing that this can produce new insights into understanding social relations and practices (Tuana, 2017, pp. 125–128; Harding, 2004, p. 25). Reemerging in the 1970s and 1980s, standpoint theory describes “a feminist epistemology, philosophy of science, sociology of knowledge, and methodology” (Harding, 2004, p. 25). In contrast to dominant approaches in science that claim objectivity, standpoint theory argues that all forms of knowledge are “socially and politically situated” (Harding, 2004, p. 39), and that specific “social locations and political struggles” (p. 26) can actually enrich the production of knowledge. Further approaches to deconstruct the subject of knowledge, for instance, include U.S. Black feminist thought (Hill Collins, 2000), critical raced-gendered epistemologies (Delgado Bernal, 2002), race-based epistemologies (Almeida, 2015), and the concept of lugar de fala (Ribeiro, 2020).

Two inherent and important features of all the approaches discussed above, which are intended to deconstruct the subject of knowledge, are the centrality of power and the different subject positionings that result from social power structures. Neglecting social power structures risks falling into epistemic relativism, thus treating all knowledge claims similarly and as equally valid (see Goldman, 1994, p. 268; Durham, 1998, pp. 124–125). Considering power in analyses of knowledge demands consistent attention to intersectionality and the reality that positionality cannot always be read by one social construct alone, rather their entanglement produces complex social power relationships. Respecting power reveals that analyses of the social production of knowledge and the formulation of claims related to interpretative power are not intellectual issues, but rather political ones (Goldman, 1994, p. 275).

## The media as part of a social epistemological process

Media represent, produce, and reproduce social discourses and serve as important producers of meaning within society (Klaus and Kirchhoff, 2016, p. 529). Media content, such as news or fiction, produces “practical social knowledge” (Hall, 2012, pp. 102–103), transmitting information about different societal groups. Fürsich (2010) postulates that “contemporary mass media operate as a normalizing forum for the social construction of reality” (p. 113). Klaus and Lünenborg (2012) designate the media “as a particular form of cultural production [that] is both an engine and an actor in the processes of self-making and being-made, in which people acquire their individual, group specific and social identities” (p. 204).

As integral parts of our society, the media contribute to the creation of meaning. They construct a representation of reality that might be interpreted by viewers as a direct reflection of the real world (Hall, 2005, p. 149). At the same time, media representations are always influenced by the meanings and experiences of the reality of everyday life (Berger and Luckmann, 1966, p. 39). Therefore, media contents are always bound to and only make sense in the context of specific societal discourses (Mikos, 2008, pp. 275–281).

Analyzing media contents in relation to the (social) production of knowledge is not new. Building on Jäger (2000, p. 19, author’s translation) definition of discourse as a “flow of (social) ‘knowledge’,” every discourse-analytical investigation of media content can be counted as an endeavor to highlight the reciprocal relation between dynamics in the social production of knowledge and media representations. Some research, however, has more explicitly focused on epistemic standards in media production and resulting representations. In particular, journalism studies have seen a growing body of research in this respect. Focusing on journalistic reporting, Jäger (2000) emphasizes the media’s central function as mediator between science, politics, and everyday life, and ascribes to the media co-responsibility for the development of “societal mass consciousness” (p. 28). In contrast to fictional content, journalism promises to make “statements about social reality” (Lünenborg, 2016, p. 332; author’s translation).

A dominant focus in research dealing with epistemic standards in journalistic production of representation concerns the idea of objectivity. Objectivity is a normative ideal within professional journalism; it is aimed at mitigating the potential of the power that results from its transformative effects (Jarren and Neuberger, 2020, p. 60) and the immense interpretative power (Fricker, 2007, p. 152) that journalists have as intermediaries. Numerous scholars in media and communication have contested the idea of objectivity, questioning whether and how journalism can represent reality (Durham, 1998, p. 117; Bach, 2016, p. 15). Bach (2016), for instance, proposes the term *discursive authority* to signify that the idea of journalistic objectivity is based on a historically grown set of rules and discursive strategies that are not universal and give power to a given object they construe to be the truth (p. 45, author’s translation). Further, Alamo-Pastrana and Hoynes (2020) argue that journalistic objectivity renders invisible the inherently subjective standpoint of a unmarked, hegemonic white norm by assuming its universality. The resulting biased representations of marginalized groups are not publicly recognized.

In light of the great impact journalism has on prevailing societal knowledge, Godler et al. (2020, p. 214) explicitly propose social epistemology as a new paradigm for discussing journalistic knowledge. They argue that in this instance, social epistemology offers both “a thorough familiarity with biases and failures of obtaining knowledge, and a strong orientation toward best practices in the realm of knowledge-acquisition and truth-seeking” (Godler et al., 2020, p. 224). Further, they emphasize the role of new technologies providing “big data and algorithmic sources” (Godler et al., 2020, p. 214) that influence (journalistic) knowledge inquiries. “New socio-digital technological systems, such as search engines, recommender systems, digital archives, and social networks” offer various ways to gain knowledge and “effectively change our epistemic standards” (Godler et al., 2020, p. 222). Neuberger et al. (2019) have conceptualized the role of the Internet in changing approaches to the “generation, examination, distribution and acquisition of knowledge in public media communication” (p. 167, author’s translation). They introduce their model of a knowledge order that is subject to digital changes. These changes are reflected in increasing demands to participate in the genesis, distribution, and examination of knowledge, the challenging of epistemic authorities, and the spread of alternative approaches to knowing and knowledge (Neuberger et al., 2019, p. 169).

Digital media environments thus have the potential to challenge epistemic hierarchies that had previously been solidified in institutionalized routines and practices. Thus far, media and communication research has primarily discussed this potential to challenge dominant epistemic norms and spread alternative accounts in relation to misinformation and the erosion of a joint societal “knowledge basis” (Neuberger et al., 2019, p. 167). Although these pose a real threat to our democratic societies that cannot be neglected, I have a different focus in this article. Using the paradigm of social epistemology, I analyze the dynamics of public knowledge production within the context of a hybrid media system. Overarching questions thereby include how and where various knowledge claims are legitimized and delegitimized in different media outlets, and whether and how they come in contact with one another. The object of investigation in my analysis is the German media debate regarding racism between 2020 and 2021. I am thus specifically concerned with the construction of knowledge related to racism in a German hybrid media landscape. The next section will introduce the contextual conditions of the public engagement with racism in Germany.

## Context: (new) public engagement with racism in Germany

In international comparison, Germany has engaged in little research on racism and rather hesitantly discussed the phenomenon in public debates (Salem and Thompson, 2016; Çaglar and Sridharan, 2021, pp. 61–62). If public debates about racism occurred, they have been either focused on right-wing extremism or have often been dominated by an U.S. perspective. In contrast, Germany has constructed itself as a colorblind society that is free of racism (Salem and Thompson, 2016). Critical scholars argue that racism in Germany is often associated with the time of national socialism and connected to the “most cruel crimes against humanity” (Rommelspacher, 2011, p. 33). Thus, the dominant German perspective is that the term does not seem suited to describe everyday racist phenomena. However, this understanding neglects the fact that national socialism also used a range of daily practices to enforce its regime (Rommelspacher, 2011, p. 33). By connecting racism to German national socialism, racism in Germany is—just like national socialism—situated in the past and thus assumed to have been overcome (Messerschmidt, 2008, p. 44).

The conscious avoidance of an active engagement with *race* in Germany since WWII is regarded as a further reason for the public hesitation to address racism (Salem and Thompson, 2016, p. 13). As part of this avoidance, a widespread consensus against the use of the term race prevails in Germany (Kerner, 2009, pp. 105–119). This is justified with the argument that human races do not exist and that “the use of the term ‘race’ entails racist implications” (Barskanmaz, 2011, p. 382). The term race is said to be antiquated and extremely burdened by its history. Here, race is reduced to its use during the time of national socialism and transnational and relational connections of racist discourses and practices are neglected. The general understanding of race in Germany is thus based on the biologically connotated concept of the term (Kerner, 2009, pp. 105–119).

Terminological variations that directly replace the term racism with alternatives like xenophobia^2^ (Rommelspacher, 2011, p. 32) further illustrate assumptions that Germany is a white country that positions racialized subjects outside the German nation (El-Tayep, 1999, p. 149; El-Tayep, 2003, p. 461; Salem and Thompson, 2016, p. 5; Kilomba, 2020, p. 115). People affected by racism are thus thought to be foreign to the German context. This reflects what Bell and Cervantez (2021), drawing on Melamed (2011), refer to as official antiracisms. Although terms like diversity or multiculturalism^3^ are used, they do not promote a critical understanding of inequalities based on race. This is in keeping with van Dijk’s (1992) description of Germany as a context in which racism is denied using various strategies that construct a “dominant white consensus” (p. 89), thus excluding the knowledge of subjects experiencing racism and ultimately reproducing racism.

Public disengagement with racism in Germany was interrupted in the summer of 2020. Worldwide BLM protests following the killing of George Floyd spread all over Germany. Milman et al. (2021, p. 7) recorded 83 protest events that gathered 200,000 protesters in various German cities and towns. The protests were primarily organized and mobilized via social media, particularly on the platforms Instagram and Telegram. The organizers were usually “young Black Germans” (“many of them women”), whereas “other groups, such as African migrants and migrant or refugee self-organizations, were less centrally involved” (Milman et al., 2021, p. 8). Thereby, organization of protests led to both the foundation of new antiracist initiatives, as well as the revival of old ones. Although the protest events were related to the U.S. and the killing of George Floyd, they were usually further contextualized in Germany and thematized structural racism in the country (Milman et al., 2021, pp. 2–9).

The various protest events relating to BLM have received “extensive media coverage” (Milman et al., 2021, p. 2) and “created unprecedented visibility for Black activism,” thus calling attention to Black Germans as “invisible minority” in Germany (Milman et al., 2021, p. 7). Thereby, the protests initiated a mainstream discussion about racism in Germany that disrupted the dominant hesitation to address the topic in this country (Agar, 2020; Haruna-Oelker, 2020; Zajak et al., 2021, p. 319; Milman et al., 2021; NaDiRa, 2022, p. 13). Therefore, these protests are often seen as a “turning point” (Milman et al., 2021, p. 12). Regarding this background, within Germany, the public debate about racism after the killing of George Floyd and following BLM protests represents a new confrontation with the topic of racism. However, although coverage on BLM protests was initially “sympathetic” to the movement in Germany (Milman et al., 2021, p. 12), various social media platforms soon saw a rise in discussions concerning the alleged exclusion from traditional media of people who experience racism. In reaction, content creators started to publish formats complementing, critiquing, or countering the engagement of traditional media.

As described above (section *Knowledge and Power*) scholars from various backgrounds, including feminist, critical race, and decolonial philosophy, have examined the relation between knowledge and power. Given this article’s focus on the construction of knowledge in the German public media debate on racism, I situate my work in the paradigm of Critical Race Theory (CRT). CRT is based on ideas by “Black activist scholars—borrowing and formalizing these concepts into general descriptions that were easy to apply to related fields “(Ray, 2022, p. 4). Solorzano (1998) defines five aspects that define CRT: 1. “The centrality and intersectionality of race and racism,” 2. “The challenge to dominant ideology,” 3. “The commitment to social justice,” 4. “The centrality of experiential knowledge” and 5. “The interdisciplinary perspective” (pp. 122–123). Thereby, a central concern of CRT is to contextualize social phenomena in their complexity (Ladson-Billings, 2003, p. 11) and supplant “taken-for-granted norms around unequal binaries” (Ladson-Billings and Donnor, 2005, p. 291). Instead of reversing epistemic hierarchies, CRT fosters the acknowledgement of different ways of knowing within structures of power and domination. Critical race theorist Ladson-Billings illustrates this complex approach to truth in CRT by addressing the reader in her writing as follows:

“I ask you to recognize the “truths” your epistemology illuminates and what “truths” are simultaneously occluded by it. I ask you to keep open the possibilities of limitless thinking and innovation. I ask you to remember that in a society structured by dominance and subordination, it’s someone else’s world; we just try to explain it.” (Ladson-Billings, 2003, p. 12).

## Materials and methods

As “flow[s] of (social) ‘knowledge’” (Jäger, 2000, p. 19, author’s translation), discourses represent epistemic dynamics within social collectives. As a toolkit offered by Foucault for the analysis of social systems of power (Jäger, 2000, p. 18), discourse analysis is thus suited to the examination of the dynamics of public knowledge production within the context of a hybrid media system. Many scholars use the approach to research media content (Wiedemann and Lohmeier, 2019, p. 5). Acknowledging the critical roots of feminist, critical race theorist, and decolonial approaches in analyzing the subject of knowledge, I regard the normative framework of CDA as suitable for my research interest, as it is characterized by a focus on how “power relations are exercised and negotiated in discourse” (Machin and Mayr, 2012, pp. 4–5).

My suggested focus on epistemic practices in the analysis of media content through CDA further demands epistemic self-reflection by the researcher as well as reflection on their impact on the research project. For me, this epistemic self-reflection translates into a praxis of transparency. In addition to revealing my theoretical and methodological approaches, this includes openness regarding the source of my epistemological interest. Through my studies in social sciences and humanities, I have been socialized in democratic thinking, which demands equality and fair representation in the media and political systems. I thus view questions of representation and social inequality as democratic issues that are importantly negotiated in and through the media. My research interest in seeing how this is represented in media debates about racism is shaped by this political standpoint. Importantly, my intellectual positioning and corresponding choice of methods, terms, and interpretative frameworks have developed in a European context. To expand this view, I am aware of the importance of exchange with other epistemologies, paradigms, and contextual conditions. This research is thus one product of a still ongoing self-reflection and permanent negotiation of what it means to be critical.

To analyze the construction of knowledge about racism in German media discourse, this article focuses on political talk shows. According to Goebel (2017, p. 404), political talk in talk shows reproduces the hegemonic discourse as well as dominant and subdominant (not subaltern) perceptions. It is thus interesting to analyze which topics are debated and how, who is invited as a (legitimate) speaker, and which discourse positions and perspectives are present in a talk show. Episodes of the shows were included if the entire show or a part of it:

- thematized prejudice, hierarchies, or essentializations based on race;
- reflected on prejudice, hierarchies, or essentializations based on race;
- described the positions and experiences of people in structures of racial hierarchization;
- described causes for and consequences of prejudice, hierarchies, or essentializations based on race; or
- discussed initiatives to tackle prejudice, hierarchies, or essentializations based on race.

To acknowledge and to be able to analyze the affordances of a hybrid media system, the sample for this study is divided into three parts. The first part consists of episodes published by the five most popular (as measured by market share) German talk shows on German public television.^4^ These include the shows *Markus Lanz* (market share: 13.3%), *Anne Will* (market share: 12.5%), *Maybrit Illner* (market share: 12.4%), *Hart aber fair* (market share: 9.3%), and *Maischberger* (market share: 9%; market shares indicate the status as of 2018; Weidenach, 2020). In addition to contributions on television, German public broadcasters also offer audiovisual formats on social media platforms. This content is produced by *Funk,* a content network that ARD and ZDF started in October 2016 with the intention of reaching audiences between the ages of 14 and 29 with their contributions (Granow, 2020, p. 363). Although social media platforms are among Funk’s major distribution channels, the network is also engaged in productions that are broadcast on public television. The second part of the sample comprises talk show episodes matching the above-mentioned sampling criteria that are published by and in collaboration with Funk. The last part of the sample consists of *non-institutionalized talk shows on social media*. With the intention of complementing public broadcasting’s engagement with racism, individual actors produced their own talk shows on various social media platforms. These talk shows include perspectives on racism that—in the view of their producers—were not represented in hegemonic mass media content. Three prominent examples of these talk show formats were selected for this part of the sample.

The sample comprises talk shows episodes that fall into the three parts that were published over the period from 25 May 2020 through 11 November 2021. The beginning of the sample period marks the killing of George Floyd; the end was decided on during the sampling process and justified by data saturation, as well as the feasibility of analysis (see Meyen, 2013, pp. 54–55). To select episodes for the first two parts of the sample, I used a webpage^5^ that stores information about content published on German television as well as the public broadcasters’ archives. For selection of the non-institutionalized shows, I relied on examples that were shared in my own social media environments. Table 1 lists the distributions comprising the resulting sample.

**Table 1:** Sample.

Sample part	Number of show episodes selected
Public television	18
Funk	10
Non-institutionalized content	16
Total	44

## Results

The analysis revealed three prominent patterns through which knowledge about racism and legitimate speaker positions on the topic were constructed within the shows: (1) performances of a rational and equitable exchange of opposing epistemic positions, (2) performances of counter-hegemonic positionality in communal exchange, and (3) performance of a rational exchange of embodied knowledge. Below, I describe each of these approaches before discussing their implications together. To illustrate how these approaches were represented in the shows I selected one example per approach. These examples are so-called typical cases. Thus, they serve as representatives for other show episodes in the sample that followed the same approach to construct knowledge about racism.

### Representing normative universality: performing the rational and equitable exchange of opposing epistemic positions

The first pattern describes the performance of a rational exchange between opposing epistemic perspectives within some of the shows analyzed. In this pattern, the talk show format pushes the participants into the roles of opposing positions and thus produces them as standpoints in the discourse. Only the show host remains unmarked and performs the role of the critical mediator between the opposing positions. One example of this is represented by an episode of the talk show *Markus Lanz* broadcast on 17 June 2020, that presents a discussion between former politician and lawyer, Wolfgang Bosbach, and political scientist, Joshua Kwesi Aikins, on racism within the German police. In the beginning of the episode moderator Markus Lanz introduces Joshua Kwesi Aikins and Wolfgang Bosbach: “We are very much looking forward to an interesting discussion between these two […]. The question is: ‘Is there such a thing as latent racism in the police force?’” (Lanz, 2020, at 2:23–3:44, author’s translation).

By sketching out their perspectives and announcing their exchange as “interesting,” Lanz suggests that they represent different—maybe even countering positions—on the topic of racism. The representation reflects an inherent feature of political talk shows, which is the representation of antagonistic perspectives on a topic. The introduction of Bosbach and Aikins is further continued in fade-in chyrons that detail Bosbach’s professional activity as a lawyer and his work for the German government, whereas Aikins’ involvement in antiracist change initiatives as an activist is emphasized. Host Markus Lanz is not described by any fade-in chyron; in contrast to Aikins and Bosbach, he is not explicitly associated with any position in the show. This supports his performance as a neutral moderator who asks critical, investigative questions to ensure a deep understanding based on which the audience can build informed opinions on the subject matter.

The spatial set up and camera action add to the performance of the participants’ different positionings. Showing the participants alternately in quick cuts mimics the course of their conversation and visualizes the tension lines (*Spannungslinien*) between the speakers (see Holly et al., 1986, p. 188). The camera thereby takes in the same angles for the different speakers and captures their reactions to what is being said (e.g., nodding or head shaking). The alternating camera angles and perspectives thereby represents the different perspectives present in the show (Holly et al., 1986, p. 180). By staging the speakers identically, the camera action implies that they are engaged in an equal exchange of knowledge independent of societal power dynamics.

Although these aspects represent general features of a talk show, they also significantly influence the construction of knowledge and legitimate speaker positions on the topic of racism. As the show host and moderator, Lanz is presented as the mediator for a diverse audience.^6^ Through his questions, and in his role as a critical moderator and host, Lanz has substantial epistemic power in the show. He can direct the discussion through further questions, interruptions, and reinterpretations of what is being said. Importantly, he influences what is regarded as common sense and which statements require further explanation, thus marking the boundaries of un/expected prior knowledge the audience should have.

The first sequence of the conversation serves as a suitable example to illustrate the interpretative power Lanz has in the show. He starts by asking Aikins about his assessment of racism within the German police. When Aikins shares a detailed account of structural racism within the force, Lanz interrupts to ask for a specific example and Lanz brings up Görlitzer Park in Berlin, which is a known space for drug dealing in the city. Lanz shares his observation that it is “[…] of course often people from African countries […]” who sell drugs in the park (Lanz, 2020, at 59:15–59:21, author’s translation). He asks whether it can be regarded racist if policepersons, based on their learning, check people they regard to be of African descent for drugs (Lanz, 2020, at 59:21–59:42, author’s translation). Lanz here constructs the consensus that people dealing drugs in Görlitzer Park are usually people of African descent. He does not further reflect on this statement by, for instance, asking why people of African descent might be overrepresented in Görlitzer Park. He thus presents an abridged description of the situation in this location, one that conceals structural processes in the constitution of subjects. Instead of a critical reflection of the situation, he reinforces the hegemonic stereotype of the criminal, non-white migrant (e.g., Said, 1979; Mercer, 1999, p. 437; Jäger, 2000).

To summarize, within this set-up the equitable exchange of different discourse positions is performed. The representation of the different positions embodied by the participants, who are shown in the same camera angles and shots, implies a balanced discussion between equally recognized discursive standpoints. Durham (1998, pp. 124–125), with reference to journalistic objectivity, mentions the danger of representing epistemic relativism in treating all knowledge claims as equally valid in the name of balanced reporting. Rather than critically questioning the participants’ positions in German discourse regarding racism, the show’s performance of an equitable exchange obscures epistemic power structures. Moreover, the show represents the amplification of a “dominant white consensus” (van Dijk, 1992, p. 89). In “perpetuating as common-sensical notions of who ought to be treated as authoritative” (Reese, 1990, p. 394), the show reproduces these epistemic power structures. The show thus represents a notion of universality that is based on prior normative assumptions of legitimate knowledge and legitimate speaking positions. Although purporting to perform a critical and universal evaluation of the topic, these assumptions are perpetuated. I thus refer to this performance as a representation of *normative universality*.

### Redefining the subject of knowledge: performing counter-hegemonic positionality in communal exchange

The second pattern is characterized by redefinition of what constitutes legitimate knowledge in comparison to hegemonic norms of rationality, objectivity, and universality. The show *Sitzplatzreservierung* serves as a suitable example to describe this pattern. *Sitzplatzreservierung* is a series of fifteen videos published on Instagram via the personal accounts of Aminata Belli and Hadnet Tesfai. Within these videos Belli and Tesfai talked to Black Germans about their perception of racism in Germany. Their guests were usually known public figures, such as singers, authors, comedians, or actors. Even though the two are both journalists in Germany and work for public broadcasting institutions, they state that *Sitzplatzreservierung* was produced independently of these. They state that their first episode on June 3, 2020, was published in response to an episode of the German public television talk show *Maischberger*, which had revolved around racism and been publicly criticized for only inviting white discussants.

One of the characteristics of *Sitzplatzreservierung* is its approach to whom the show regards as being a legitimate speaker. Rather than claiming a universal, neutral perspective, it foregrounds experiential knowledge, and the speakers’ personal social locations are emphasized both visually and in how they speak. This is illustrated in the following example in which German rapper Ahzumjot shares his experience of being a Black man in Germany:

“[…] I caught myself in […], at an interview or in applying for a flat – no matter where I appeared […] I caught myself in wanting to seem particularly German. In articulating myself particularly German, also in dressing myself a bit differently […] – I do not want to cause a fuss, put stupidly. […] I can express myself as German as I want […] I can dress myself as I want. […] I will always be different.” (Ahzumjot, 2020 [X], at 26:05–43:32, author’ translation).

Here, Ahzumjot elaborates on his personal experience of the notion that being German equals being white (see El-Tayep, 1999, p. 149; El-Tayep, 2003, p. 461). As such the appearance of his body on screen, together with his elaboration serves to illustrate his positioning outside a white norm. Importantly, there is a focus on Ahzumjot’s emotional processing and subjective perception of the situation. I regard this strong personalization and linkage to experience as a form of embodiment. The knowledge shared in *Sitzplatzreservierun*g is thus thought to be embodied and influenced by social locations. This understanding of legitimate knowledge allows Ahzumjot to challenge the hegemonic, assumed neutral view that racism is nonexistent in Germany and reveals this view as one based on an unmarked, white hegemonic norm.

Further, the hosts of *Sitzplatzreservierung* explicitly articulate their standpoint in opposition to their perception of a German (media) mainstream. Within the following quote, Tesfai explains the intention behind the creation of the Instagram live series:

Tesfai: “[…] maybe we can shortly […] talk about, why we chose this name.

[*Sitzplatzreservierung* (seat reservation)]. This was of course based on this “seat at the table” and that we, so to say, created our own seat here. But do you know what someone told me today? That this [the name] reminds him a bit of Rosa Parks. And I was like: Not the worst association I would say!”

Belli: “Yes, definitely.” (Belli and Tesfai, 2020a, at 1:36–2:03, author’s translation).

Referring to the public debate about racism in German traditional media, *Sitzplatzreservierung*, according to Tesfai, was intended to represent a space where subjects experiencing racism could share their accounts of it. Drawing on U.S. American civil rights activist Rosa Parks, who famously claimed a physical bus seat in defiance of white rule, symbolically attached an activist intention to *Sitzplatzreservierung*. Viewing themselves in the role of Rosa Parks, *Sitzplatzreservierung* thus represents a counter position against hegemonic norms and standards that are influenced by white domination. Referring to Rosa Parks further put *Sitzplatzreservierung*—and by extension its creators—in a subordinate position.

The counter position of *Sitzplatzreservierung* was rendered even more by explicit statements distancing the format from traditional media contents. In particular, German public television often served to represent a public debate on racism in Germany that does not sufficiently include the perspectives of people affected by racism. In contrast to this, *Sitzplatzreservierung* claimed to offer a more holistic approach to racism by including the multifaceted perspectives of Black subjects in Germany. The counter position that *Sitzplatzreservierung* represents to German public television mainstream is thus redefined as the perspective that represents the actual lived reality of people experiencing racism in Germany. The distinction from other media content further forms a referential strategy for group affiliation (Hart, 2010, p. 49), in that *Sitzplatzreservierung* is characterized by a strong performance of stranger sociability (Warner, 2002, pp. 86–87) and community. This becomes apparent, for instance, when Tesfai defines the format’s target audience:

Tesfai: “[…] We address a Black audience. And anyone else who’s listening and takes something out of it: cool. But we first and foremost think of each other and our conversation partners. However, we of course know that this is not happening in a vacuum space.” (Belli and Tesfai, 2020b, at 13:34–14:32, author’s translation).

The series thus constructed an imagined community of Black people in Germany who are connected through their experiences with racism, and assumed intimate relations to other Black people, in turn, formed a culture through which to connect to strangers via *Sitzplatzreservierung*. The redefinition of the subject and the sharing of embodied knowledge as legitimate, knowledge-producing praxis, as well as the counter position to the mainstream, thus served as the basis for an assumed community and high levels of sociability among strangers by way of *Sitzplatzreservierung*. Simultaneously, this assumed community, based on sharing of experience and representing counterhegemonic status, was constitutive of membership in *Sitzplatzreservierung*.

### Redefining the subject of knowledge in normative structures: performing the rational exchange of embodied knowledge

The final pattern is not characterized by a homogenous account on how to construct legitimate knowledge and speaker positions regarding racism, but rather it comprises different approaches that negotiate normative standards of rationality and the acknowledgement of embodied knowledge. One example here is represented by another episode of the talk show *Markus Lanz* (17.06.2021) which focused on racism in soccer using the documentary *Schwarze Adler*^7^ as central reference point.

In one sequence of the episode, guest and former soccer player Gerald Asamoah talks about his first experience with racism during a soccer match in Cottbus in 1996:

“[…] was my first real experience with racism where I really realized that I experience hostility in soccer. It was pure hate, so when bananas get thrown at you, I was 18 at the time, […] and I have never received such hate before” (Asamoah, 2021, at 49:57–50:25).

After Asamoah says this, Lanz announces that the scene will now be shown; Asamoah signals his agreement through a nod. The next scene then shows a recording of the soccer match between the soccer clubs Energie Cottbus and Hannover in 1996, excerpted from the documentary *Schwarze Adler*. Various scenes then show moments from the match, including the players on the field and the audience in the stands. The sound captures the screams of the audience, and fast, buzzing music is added in the background. Close-up shots on the audience in the stands show the upset facial expressions of the audience, as they hold their thumbs down and shout, “Boooo.” In a later shot, soccer players on the field are shown having an argument, Asamoah is between them, and he then gets pushed by players from the opposing team. The fast-buzzing music emphasizes the heated and threatening mood expressed through these sequences. The audience’s screams and the noise in the background further convey the stressfulness of the situation. For a moment, the background noise is muted to make audible the fans who are repeatedly singing “Get the N*^8^ out” (Lanz, 2021, at 49:57–50:25). The scene ends by presenting two fans in the stands from a bottom-up perspective, in front of red fireworks, shaking the fence that separates them from the players. The subsequent shot shows Asamoah on the field burying his face in his hands. This is then followed by the same close-up shot of Asamoah’s face in the talk show studio.

The entire compilation of different scenes from the soccer match emphasizes the stress and threat described by Asamoah before the interlude. I argue that this presentation serves two purposes: First, it illustrates the experience described—the scene demonstrates Asamoah’s embodied knowledge. His interpretation of racism in soccer is thus supported and given validity. The norm of granting legitimacy to knowledge that is presented as neutral, and objective is thus disrupted by using audiovisual representations as evidence for experiential knowledge. This reflects Evans (1999) argument that Western epistemology equates knowledge with representations that “are judged according to their adequacy as ‘reflections’ of an external reality,” and that images are considered “to act as a transparent ‘relay’ to a singular originary presence which is imagined to lie behind [them]” (p. 12). The sequences thus act as exactly this transparent relay of reality, even though camera movement and sound privilege an interpretation along the lines of Asamoah’s description of threat.

Second, in visually presenting the show’s participants in their roles as soccer players experiencing racism, the documentary scenes accentuate their embodied positioning and thus pave the way for the acknowledgement of embodied knowledge in the talk show studio. In particular, the last sequence of shots shows this transition between the participants’ roles and the establishment of their embodied positioning. The fans represented, who are filmed from a bottom-up perspective, are attributed power, and the threat of the situation is signified through their aggressive rattling on the fences and the red pyrotechnic in the background, which is reminiscent of fire and explosions. The next shot of Asamoah burying his face in his hands on the soccer field implies a relation between this signified threat and this gesture of despair. The subsequent cut to Asamoah’s face in *Markus Lanz* transports this relation to the talk show studio. The focus on his body on the soccer field is thus inseparably connected to his body in the talk show studio, which renders it impossible to ignore this connection. Arguments based on embodied knowledge are thus more likely to be accepted and regarded as legitimate in this setting.

Although the soccer players’ embodied positioning in their experiences of racism is depicted in contrast to a white norm, there is no further reflection on the positioning of whiteness. The explicit marking of embodied positioning thus only happens for participants that deviate from a white norm in the show. Evans (1999) argues that images “separate the viewer from the viewed by a discontinuity in the relations between time and space” (p. 16). The focus on the embodied positioning of the participants that deviate from a white norm and their illustrated experiences in the show thus also distances them from the audience. This distancing continues in the missing bodily focus on the white participants in the show, in that the show continues the normalization of whiteness.

## Discussion

The three patterns discussed above reveal interesting dynamics between embodied representation and epistemic representation. I conceptualize embodied representation as the mere presence of subjects who are meant to represent a social group. Epistemic representation refers to the legitimate presence of different knowledges. In particular, the first pattern (*Representing normative universality: Performing the rational and equitable exchange of opposing epistemic positions*) reveals that embodied representation is not equivalent to epistemic representation. Within the example discussed above, Aikins’ claims are interrupted and delegitimized by host Markus Lanz in favor of dominant, stereotyping narratives. This highlights the importance of considering epistemic dynamics in analyses of representation: A mere focus on embodied representation risks superficial conclusions that conflate structural and individual positionings and endorse commodified representations that do not counter unequal structures. In other words, who can be seen or who is shown does not necessarily define who and what can be heard.

Conversely, the second pattern (*Redefining the subject of knowledge: Performing counter-hegemonic positionality in communal exchange*) shows an approach in which epistemic representation is specifically tied to the body in constructing embodied knowledge as the only legitimate source of knowledge about racism. The racialized body thereby forms an important marker in signaling embodied expertise and group belonging. The associated emphasis on experience reveals workings of racism in Germany that remain obscured under the first pattern’s framework of normative universality. This illustrates how using experience as a form of knowledge “recognizes that social discourses are enmeshed in lived experience and institutional and social power relations that have emotional, material and embodied consequences for individuals and for groups” (Gunaratnam, 2003, p. 7).

However, using experience to understand “social and interactional contexts” runs the risk of “maintain[ing] an essentialist view of ‘race’ and ethnicity, where experience can be seen to be wholly (pre)determined by racial and ethnic categories, that are themselves construed as unchanging ‘essences’, cordoned off from social, material and emotional relations” if one fails “to recognize the contingency and the ambivalent complexity of lived experience” (Gunaratnam, 2003, p. 6). Importantly, examples like *Sitzplatzreservierung* emphasize the heterogeneity of the communities constructed, and in their expressions, they represent a quite differentiated approach towards the constructed ingroup. Although their community construction assumes that all members of that community experience racism, the show allows for different reflections on what this experience means for the participants individually. Aspects of homogenization are rather visible in the construction of the community’s outgroup. This becomes particularly apparent in the assumption of a German media mainstream that consistently excludes people who are affected by racism. Homogenizing the outgroup constructed by German media poses a further strategy of legitimization that makes *Sitzplatzreservierung* appear more subversive.

The representation of normative universality discussed in the first pattern raises the question of how the reproduction of unequal epistemic structures can be prevented in the validation of different knowledge claims. The answer presented by approaches discussed in the second pattern involve the performance of sharing embodied knowledge. This, however, is based on the establishment of a strong collective group identity that performatively limits the targeted audience of the depicted exchange to members of that constructed group. Further, this can also run the risk of tying the correctness of a claim to the identity of the speaker and thus refute the basic social epistemological presumption that knowledge can be shared and communicated by different actors. The approaches discussed in the third and final pattern (*Redefining the subject of knowledge in normative structures: Performing the rational exchange of embodied knowledge*) combine the acknowledgement of embodied knowledge with the address of a general audience. As these approaches are distributed across platforms (social media platforms and public television), the address of a wide audience and the acknowledgement of embodied knowledge are shown to be independent of the distribution platform. Although the reading of the first two patterns might imply a causal relationship between the distribution platform and the epistemic approach to talking about racism, the approaches discussed in pattern three disprove this assumption. The fact that the two examples from the *Markus Lanz* show exhibit both the first and third patterns illustrates the variety of approaches that are possible on one distribution platform. The episode discussed in the third pattern was released exactly 1 year after the episode discussed in the first pattern, also demonstrating that the same show format can change their epistemic approach over time.

## Conclusion

Following elaborations on the social dimensions of knowledge production and inequalities within them, I introduced the media as part of a social epistemological process. Within this framework, I presented my analysis of the German public media debate regarding racism after the killing of George Floyd and following the BLM protests in the summer of 2020. By means of CDA, I investigated talk shows about racism that were released on German public television as well as the social media platforms YouTube and Instagram. My analysis revealed three patterns for constructing legitimate knowledge and speaker positions related to racism: (1) performances of a rational and equitable exchange of opposing epistemic positions, (2) performances of counter-hegemonic positionality in communal exchange, and (3) performances of a rational exchange of embodied knowledge.

My approach allowed me to focus the delicate negotiation of various power structures within the construction of knowledge about racism. Dominant approaches to producing rational knowledge risk rendering dominant nuances in social inequalities invisible by focusing on the perspective of an invisible norm. In contrast, acknowledging this deficit raises the question of how well these nuances can be represented with a sole focus on embodied knowledge. Having each nuance represented by an individual runs the risk of again reifying thinking regarding embodied representation and a mere phenotypical understanding of representation. Representation remains a delicate practice that must be constantly questioned. Castro Varela et al. (2018, p. 270) thus call for viewing representation as a parrhesian praxis as defined by Foucault: “More precisely, parrhesia is a verbal activity in which a speaker expresses his personal relationship to truth, and risks his life because he recognizes truth-telling as a duty to improve or help other people (as well as himself)” (Foucault, 1985, p. 5).

Representation thus must always appear self-critical and critical of power at the same time (Castro Varela et al., 2018, p. 270). The self-positioning of show participants, even in the face of the obstacles of this practice, has proved to be one tool that allows movement towards this critical evaluation.

With this article, I offer two main contributions: (1) By conceptualizing the media as part of social epistemological process, I illustrate how social epistemology can be a promising paradigm for the study of media communication. This conceptual innovation allows for a nuanced and critical analysis of how inequalities can be (re)produced in media representation under the conditions of a hybrid media system. (2) I offer the first in-depth analysis of the German public media debate about racism to illustrate my conceptual approach. My results offer critical perspectives on media representation that can inform both media and communication scholarship as well as media practitioners.

## Data Availability

The raw data supporting the conclusions of this article will be made available by the authors, without undue reservation.

## Footnotes

^1^However, epistemic injustice often works as umbrella term for a multitude of concepts describing inequalities in epistemic dynamics.

^2^German terms stated in the original quotation include “Ausländerfeindlichkeit, Fremdenangst oder Fremdenfeindlichkeit” (Rommelspacher, 2011, p. 32).

^3^Hall (2021a) explained that multiculturalism and racism “proceed hand in hand,” adding “other dimensions of racialized otherness” to already existing “racialized repertoires” (Hall, 2021a, pp. 404–405). Moreover, Hall (2021b) described different workings of multiculturalism. “Commercial multiculturalism,” for example, “exploits and consumes difference in the spectacle of the exotic ‘other’” (Hall, 2021b, p. 410).

^4^Made up of ARD, ZDF, and Deutschlandradio, the public broadcasters are financed by fixed contributions paid by each household every month by law. Public broadcasting stations do not pursue political or economic interests but are meant to provide access to information, education, entertainment, and culture to every person living in Germany. Their reporting is supposed to be objective and independent and to serve as basis for people’s opinion building (Mitteldeutscher Rundfunk [MDR], 2018).

^5^ https://www.etwasverpasst.de/

^6^As a show produced by Public Service Broadcasting (öffentlich-rechtlicher Rundfunk), *Markus Lanz* is a public and meritorious good that can be accessed by various groups across German society (Gundlach, 2020, p. 1513).

^7^The documentary *Schwarze Adler* tells the story of soccer players who have been part of the German national team and have experienced racism. The documentary retraces their experiences with racism. It was produced by ZDF and first publicly aired on 18 June 2021.

^8^The N-word is fully pronounced in the scene.
